# The Efficacy of Erlotinib Versus Conventional Chemotherapy for Advanced Nonsmall-Cell Lung Cancer: A PRISMA-Compliant Systematic Review With Meta-Regression and Meta-Analysis

**DOI:** 10.1097/MD.0000000000002495

**Published:** 2016-01-15

**Authors:** Hu Ma, Xu Tian, Xian-Tao Zeng, Yu Zhang, Yi Wang, Fei Wang, Jian-Guo Zhou

**Affiliations:** From the Department of Oncology, Affiliated Hospital of Zunyi Medical University, Zunyi, P.R. China (HM, YZ, YW, FW, JGZ); Center for Translational Medicine, Zunyi Medical University, Zunyi, P.R. China (JGZ, HM); Graduate College, Tianjin University of Traditional Chinese Medicine, Tianjin, P.R. China (XT); School of Nursing, Tianjin University of Traditional Chinese Medicine, Tianjin, P.R. China (XT); Center for Evidence-Based and Translational Medicine, Zhongnan Hospital of Wuhan University, Wuhan, P.R. China (XTZ); Center for Evidence-Based and Translational Medicine, Wuhan University, Wuhan, P.R. China (XTZ).

## Abstract

Non-small-cell lung cancer (NSCLC) is the leading cause of cancer deaths. Erlotinib is the first-generation epidermal growth factor receptor-tyrosine kinase inhibitors (EGFR-TKIs), the National Comprehensive Cancer Network (NCCN) guidelines recommend it as a first-line agent in patients with sensitizing EGFR mutations.

We conducted a meta-analysis to compare the efficacy of erlotinib and chemotherapy for advanced NSCLC, and evaluated the efficacy of them to provide references for further clinical practice and research.

PubMed, EMBASE, CBM, CNKI, *WanFang* database, The Cochrane library, and Web of Science, as well as abstracts presented at ASCO conferences and ClinicalTrials.gov were searched to identify relevant studies. HR with 95% confidence intervals (CIs) for progression-free survival (PFS) and overall survival (OS), relative risk (RR) with 95% CIs for objective response rate (ORR) and 1-year survival rate (OSR) were all extracted. If the I^2^ was ≤40%, then the trial was considered to be heterogeneous, and a fixed-effects model was selected. Otherwise, a random-effects model was used. Meta-regression and sensitivity analyses were conducted to determine the possible heterogeneity causes and to further identify the influence of the various exclusion criteria on the overall risk estimate.

The pooled analysis demonstrated a PFS HR of 0.93 (95% CI = 0.73, 1.19) for erlotinib versus chemotherapy and an ORR of 18.43% versus 22.07%, respectively. The OS HR was 1.02 (95%CI = 0.93, 1.12). The HRs for PFS estimated based on 10 trials involving 1101 patients were 0.22 (95% CI = 0.15, 0.29) and 1.27 (95% CI = 1.04, 1.48) in EGFR mutation-type and wild-type patients, respectively. The HRs for OS calculated from 4 studies including 681 participants were 0.83 (95% CI = 0.61, 1.05) and 0.86 (95% CI = 0.68, 1.04) in EGFR mutation-type and wild-type patients, respectively. The 1-year survival rates were 31.31% and 32.41%, respectively.

Overall, the present meta-analysis suggested that erlotinib did not improve the ORR, PFS, OS or the 1-year survival rate for whole patients. However, erlotinib could benefit patients with EGFR mutation in terms of PFS, but the OS does not benefit from it for these patients. Further studies of erlotinib for these subgroup patients are warranted.

## INTRODUCTION

Lung cancer is the leading cause of cancer deaths in China and over the world, and nearly 1 million new cases are expected annually by 2025.^1–3^ Non-small cell lung cancer (NSCLC) accounts for more than 85% of all lung tumors.^4^ Approximately 60% of diagnosed NSCLCs are in the terminal stage. The median overall survival of patients treated with first-line chemotherapy ranges from 7 to 12 months.^5^ Second- and third-line chemotherapy treatments have been used to further increase survival rates. Despite the use of a combination of all current therapies, patient survival remains unoptimistic.^6^

In 2013, the Food and Drug Administration (FDA) approved erlotinib (Tarceva®) as a first-line treatment for metastatic NSCLC patients with EGFR mutations.^7^ The NCCN also recommended erlotinib as a first-line therapy in patients with sensitizing EGFR mutations. However, it did not recommend that erlotinib be given as first therapy for patients with a negative or unknown EGFR status. As a second-line therapy, erlotinib is superior to the best available supportive care. However, as a third-line therapy, the efficacy of erlotinib is uncertain.^8^

Numerous clinical trials have been developed to evaluate the efficacy of erlotinib in the treatment of advanced NSCLC, either in combination with chemotherapy or alone; however, consistent results have not been identified, and our meta analysis showed that erlotinib combined with CT could increase PFS and objective response rate, but not benefit OS,^9^ our another meta analysis disclosed that erlotinib could decrease the incidence of neutropenia and leukopenia in patients with advanced NSCLC undergoing erlotinib regardless of whether combined with CT or not.^10^ In recent years, many published meta-analyses have been focusing on EGFR-TKIs for NSCLC^11–14^; however, all 4 studies explored a combination of EGFR-TKIs rather than the effects of single agent. However, some studies reported different antitumor activities and favorable toxicities for various oral EGFR-TKIs.^15^

Therefore, a pooled analysis of the currently available studies that were restricted to patients who used erlotinib alone compared with other chemotherapy, which may provide relevant information for the treatment of patients with advanced NSCLC, was performed to evaluate the efficacy of erlotinib compared with chemotherapy. Additionally, we performed meta-regression and subgroup analyses according to the treatment period, ECOG-PS, gender, EGFR mutation status, and smoking status. We also comprehensively appraised the quality of the evidence with GRADEpro to facilitate clinical decision-making.

## METHODS

Ethical approval and patient written informed consent are not required due to that this is a systematic review and meta-analysis of previously published studies. This study was performed in accordance with Preferred Reporting Items for Systematic Reviews and Meta-Analyses (PRISMA) statement.^16^ The protocol was published by Centre for Reviews and Dissemination PROSPERO (Registration No. CRD42014010347).

### Search Strategy

Eligible trials were identified by electronically searching PubMed, EMBASE, ISI Web of Science (ISI), and The Cochrane Central Register of Controlled Trials (CENTRAL) with the following terms: (“non-small-cell lung carcinoma” OR “non-small cell lung cancer”) AND (“Erlotinib” OR “Tarceva”) (from inception to December 25, 2014, updated at October 28, 2015). The PubMed search strategy is summarized in Appendix 1. The abstracts indexed in ASCO and ESMO and search engines, including Baidu (Chinese), Google Scholar and DXY.com (Chinese), were also searched to include any potential studies. The reference lists of the included studies were also manually evaluated to improve the recall ratio, and precision ratio. No language restriction was imposed.

### Selection Criteria

Using the PICOS acronym (population, intervention comparison, outcome, and study design), the following inclusion criteria were identified: *Population*: all the patients who were diagnosed as advanced NSCLC using pathology and cytology tests were eligible for the systematic review. The patients’ nationality was not limited, and the patients did not have any other complications, such as serious cardiopulmonary diseases and other severe basic diseases. *Interventions and comparisons*: the intervention is erlotinib alone, the comparison is conventional chemotherapy regardless any regimens or cycles. *Outcomes*: the overall survival (OS), objective response (ORR), progress-free survival (PFS), and 1-year survival rate (OSR) were evaluated.

### Data Extraction

Two reviewers (Jian-Guo Zhou and Yu Zhang) independently screened the titles and abstracts to exclude studies that failed to meet the inclusion criteria, and the full texts of the remaining studies were subsequently reviewed. Finally, data extraction was conducted with a premade data extraction form to collect information about the authors, the populations studied, publication year, country, and detailed information regarding the PICOs. The formula recommended by Tierney et al was adopted to calculate the corresponding HR of the missing data.^17^ Kaplan–Meier curves were produced with the Engauge Digitizer, version 4.1 (free software downloaded from http://sourceforge.net/) if the available data is not directly shown.^9^ Yu Zhang performed the data extraction and entry, and Jian-Guo Zhou examined the data. Any disagreement between the researchers concerning trial eligibility was resolved by consulting a third reviewer (Xu Tian). Each trial included in the study was independently evaluated for bias assessment risks according to the Cochrane Collaboration's tool by 2 reviewers (Fei Wang and Yi Wang).^18^

### Level of Evidence

The GRADE profiler software (version 3.6) (available at: http://www.gradeworkinggroup.org/) was used to evaluate the level of evidence, and an evidence profile was developed to reveal the summary results.

The GRADE system identified the following four rating grades of evidence quality^19^: *High*: further research is very unlikely to change our confidence in the effect estimate; *Moderate*: further research is likely to have an important impact on our confidence in the effect estimate and may change the estimate; *Low*: further research is very likely to have an important impact on our confidence in the effect estimate and is likely to change the estimate; and *Very low*: any effect estimate is very uncertain.

### Statistical Analysis

All data were pooled using STATA, version 12.0 (Stata Corp., College Station, TX). The effect size indicators, including HR, risk ratio (RR) and corresponding 95% CIs, were calculated. Heterogeneity among the included studies was evaluated with I^2^ statistics. I^2^ of 40%, 70%, and 100% were used to represent low, moderate, and high heterogeneity, respectively. If the I^2^ was ≤40%, then the trial was considered to be heterogeneous, and a fixed-effects model was selected. Otherwise, a random-effects model was used.^20^ Meta-regression and sensitivity analyses were conducted to determine the possible heterogeneity causes and to further identify the influence of the various exclusion criteria on the overall risk estimate. The influence of individual trials was also investigated with the leave-one-out cross validation method to test the robustness of the primary outcomes.^21^ Publication bias was assessed graphically using funnel plots and regression tests, according to the method reported by Egger,^22^ and by the Begg test.^23^ A *P*-value < 0.05 was considered statistically significant.

## RESULTS

**FIGURE 1:**
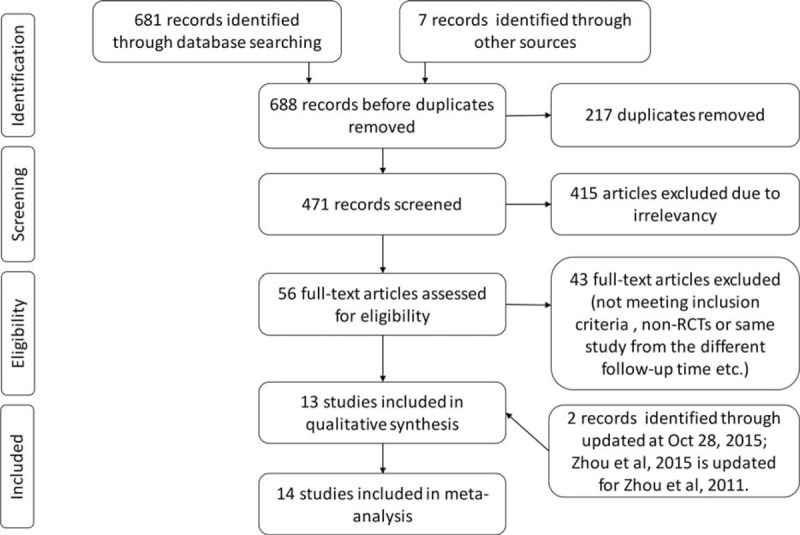
Flow diagram of the study details.

A total of 688 unfiled titles and abstracts were identified in the initial search, and 14 studies,^13,24–39^ which involved a total of 3559 participants, met the inclusion criteria and were thus included in the final analysis. A flow diagram of the literature that was searched and evaluated is presented in Figure 1.

All the eligible studies were published between 2008 and 2015. In total, 13 trials provided PFS outcomes and 1 study reported the tumor progression time.^29^ The objective response rate and overall survival outcomes were available in 10 and 13 trials, respectively. The main characteristics of the included studies are recorded in Table 1.

**TABLE 1:**
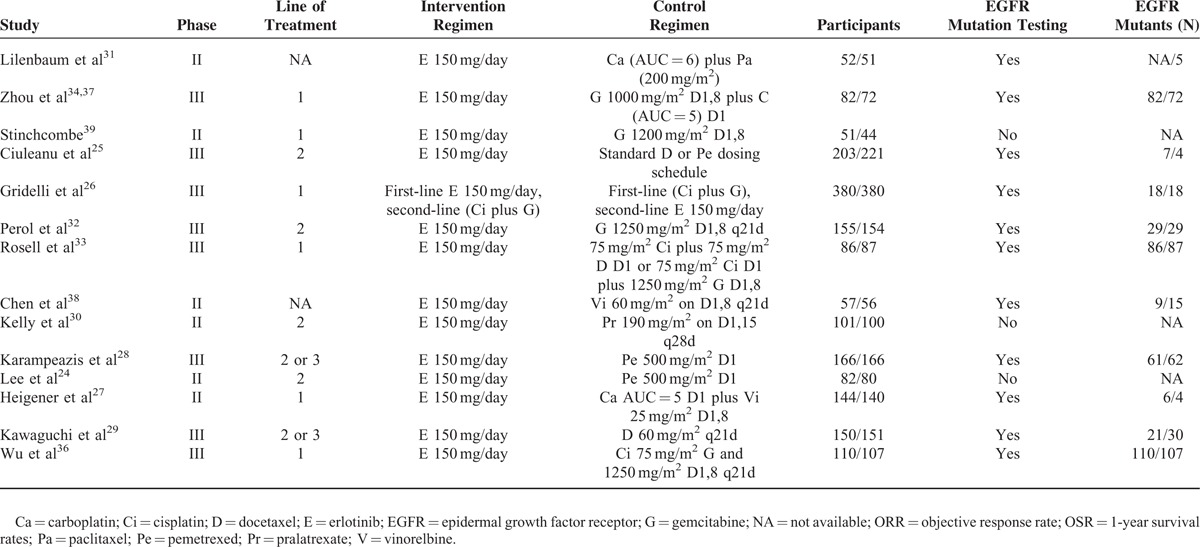
The Main Study Characteristics

**FIGURE 2:**
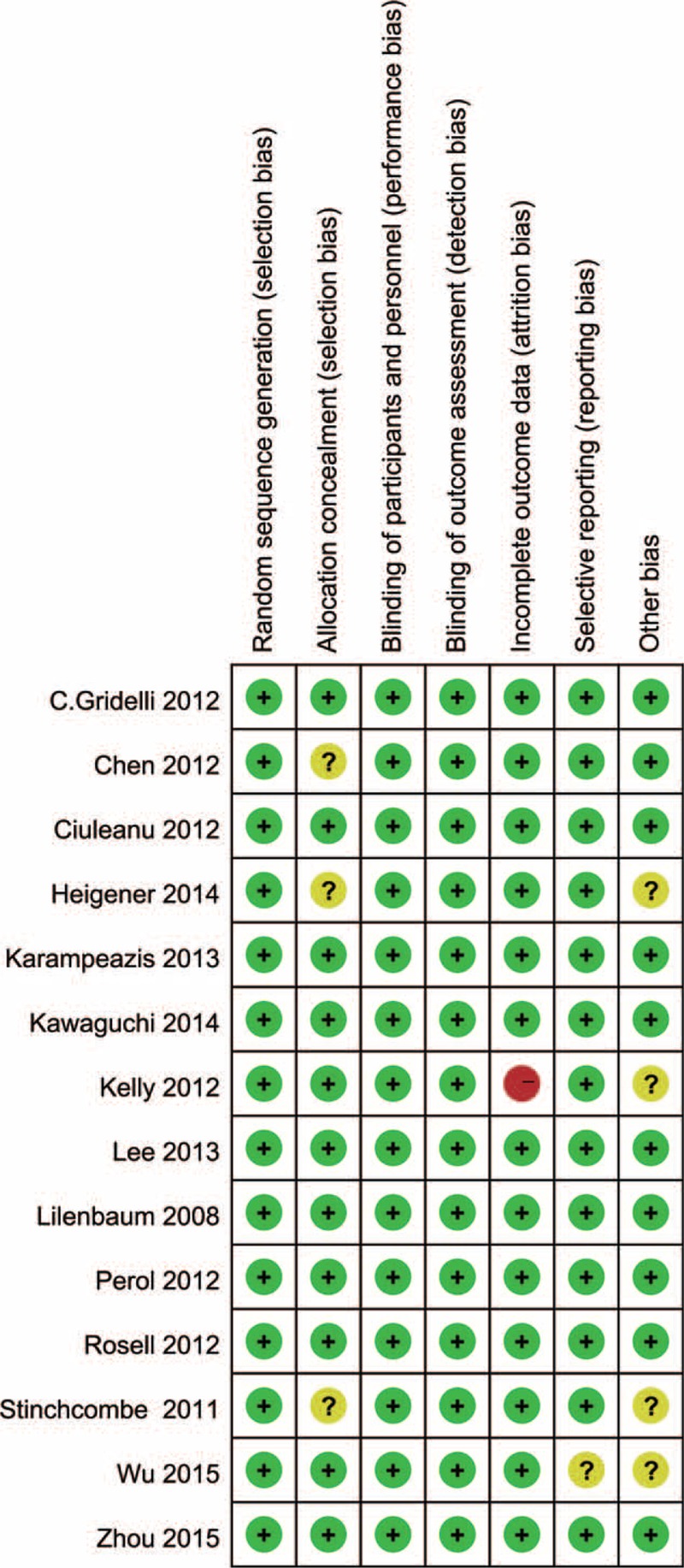
Appraisal of risk of bias of the included trials using the Cochrane risk-of-bias tool.

All 14 trials were open-label. Random sequence generation and allocation concealment were performed adequately in most of the trials. However, 1 trial did not describe the reasons for incomplete outcome data.^30^ Under the assumption that the PFS outcome might not differ from the progression time, the PFS data were used and pooled.^13^ The blinding method was unclear for all the trials. However, it was unlikely to affect the quality assessment. Three references^33,34,38^ had small sample sizes and eventually included fewer than 150 cases. The overall methodological quality of the included trials was generally good and fair (Figure 2).

### Objective Response Rate (10 Trials, 2560 Patients)

**FIGURE 3:**
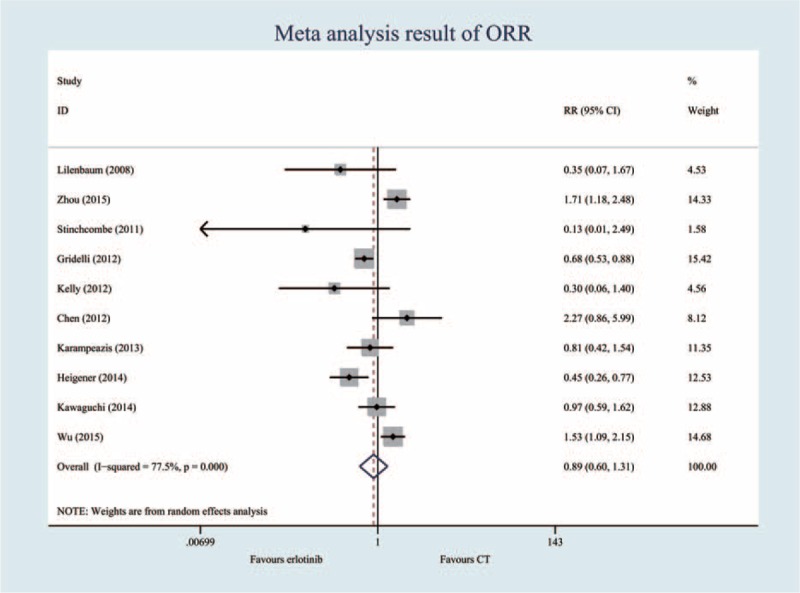
Meta-analysis results of the objective response rate.

According to the heterogeneity test, the I^2^ was 77.5%, and the *P*-value was less than 0.05. Thus, a random-effects model was selected. The pooled RR for ORR showed that there were no significant differences between the erlotinib regimen and chemotherapy regimen groups (RR = 0.89; 95% CI = 0.60, 1.31, *P* = 0.560) (Figure 3, Table 2).

**TABLE 2:**
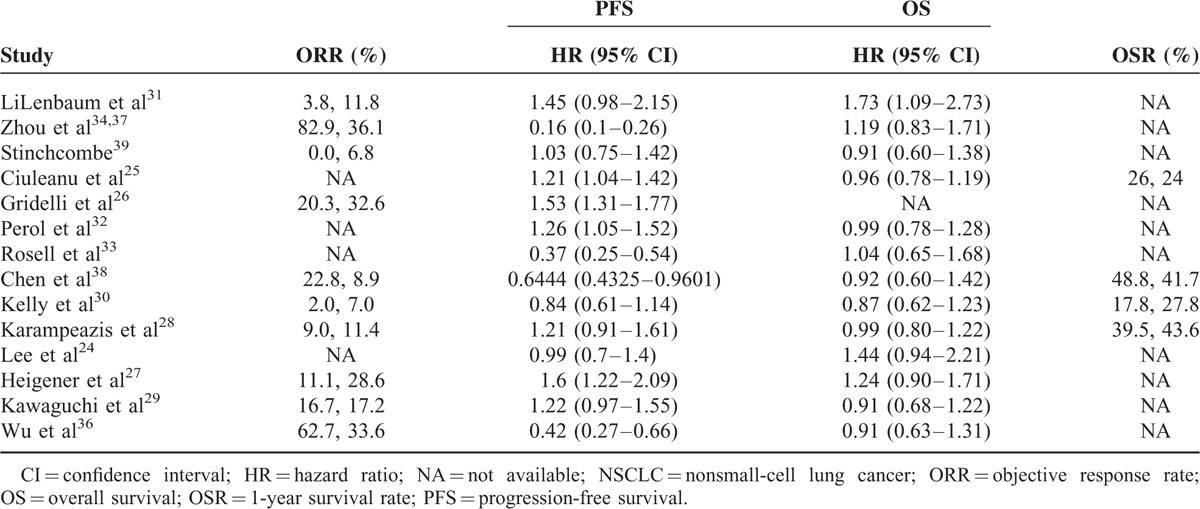
The Overall Survival Result of Erlotinib Versus Conventional Chemotherapy for Advanced NSCLC

### Progression-Free Survival (14 Trials, 3559 Patients)

**FIGURE 4:**
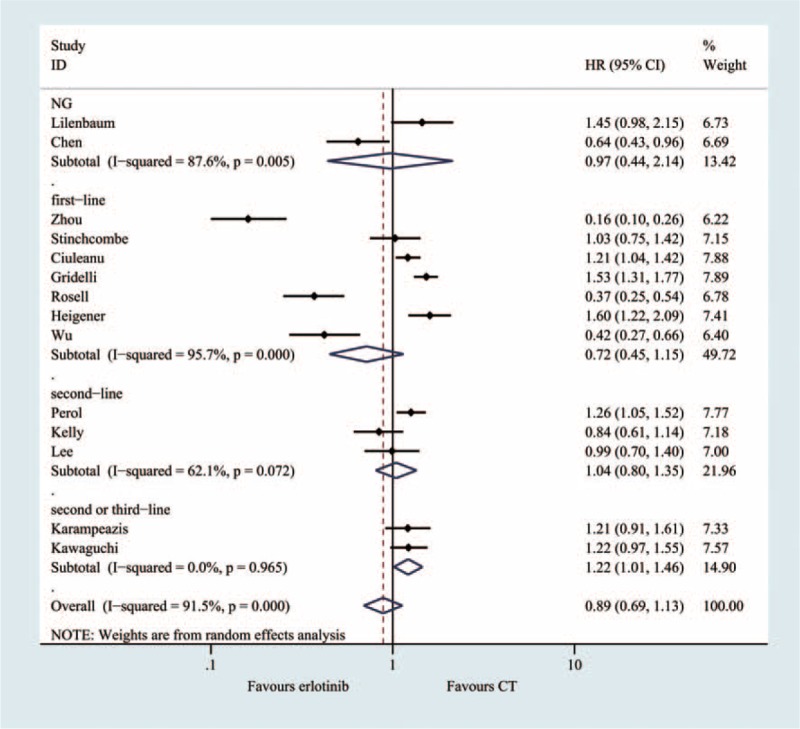
Meta-analysis results of the progression-free survival.

The PFS of the erlotinib arm ranged from 1.6 to 13.1 months, and the PFS of the chemotherapy arm ranged from 1.2 to 5.2 months. The meta-analysis showed that the pooled HR was 0.98 (95% CI = 0.69, 1.27; *P* = 0.330), without statistical significance when the erlotinib regimen patients were compared with the chemotherapy regimen patients (Figure 4, Table 2).

### Overall Survival (13 Trials, 2868 Patients)

**FIGURE 5:**
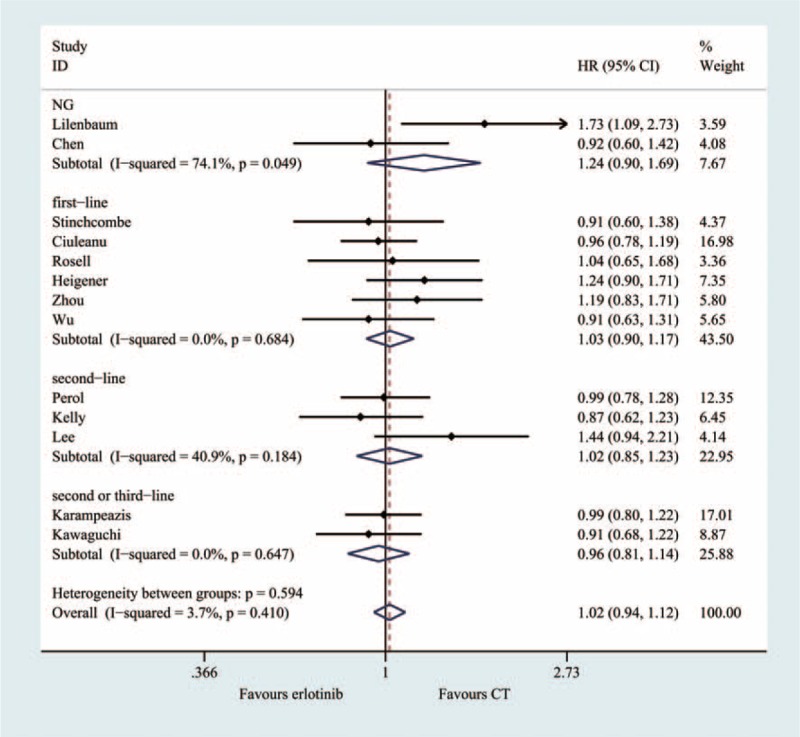
Meta-analysis results of the overall survival.

A total of 13 RCTs were included in the meta-analysis that was used to evaluate overall survival. The heterogeneity test indicated that a fixed-effect model could be selected (I^2^ = 3.7%, *P* = 0.410). The pooled results of the meta-analysis showed that there was no significant difference between the 2 groups (HR = 1.02; 95% CI = 0.94, 1.12; *P* = 0.609) (Figure 5, Table 2).

### One-Year Survival Rate (4 Trials, 1070 Patients)

**FIGURE 6:**
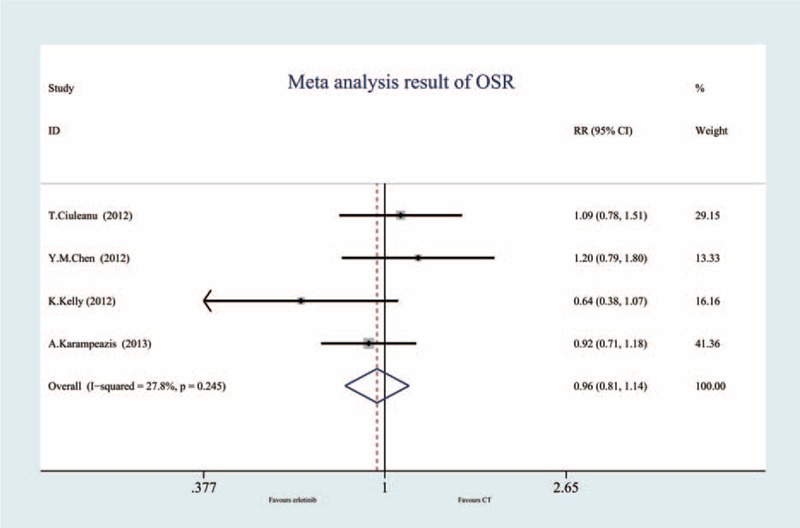
Meta-analysis results of the 1-year survival rate.

Four RCTs evaluated the 1-year survival rate. There was no significant heterogeneity (I^2^ = 27.8%, *P* = 0.245), therefore, a fixed-effect model was used. The result of the meta-analysis suggested that there was no significant difference between the erlotinib and conventional chemotherapy groups (RR = 0.96; 95% CI = 0.81, 1.14, *P* = 0.632) (Figure 6, Table 2).

### Subgroup Analyses

**FIGURE 7:**
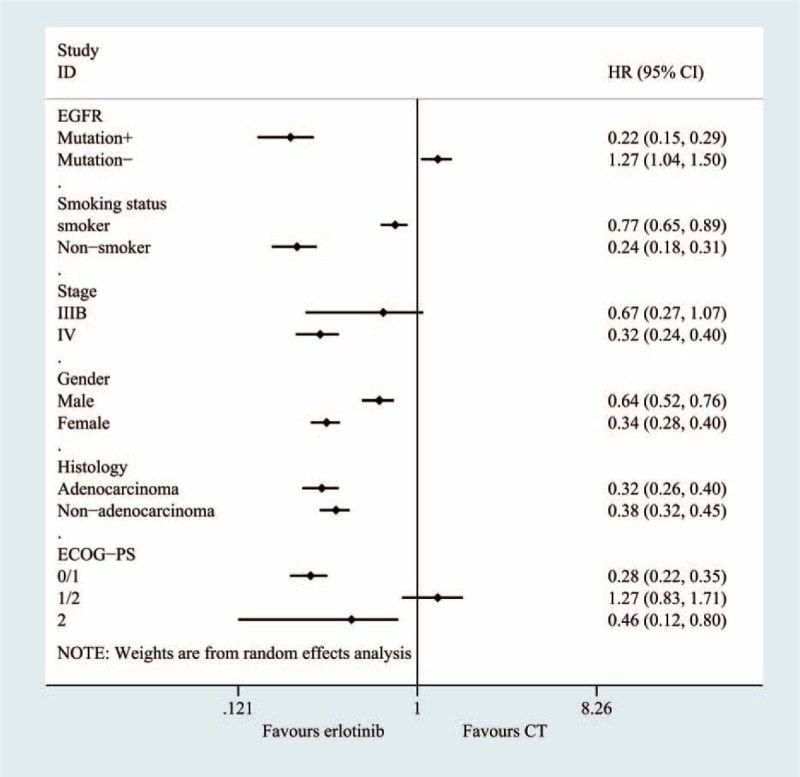
Subgroup and meta-regression analyses of the PFS.

**FIGURE 8:**
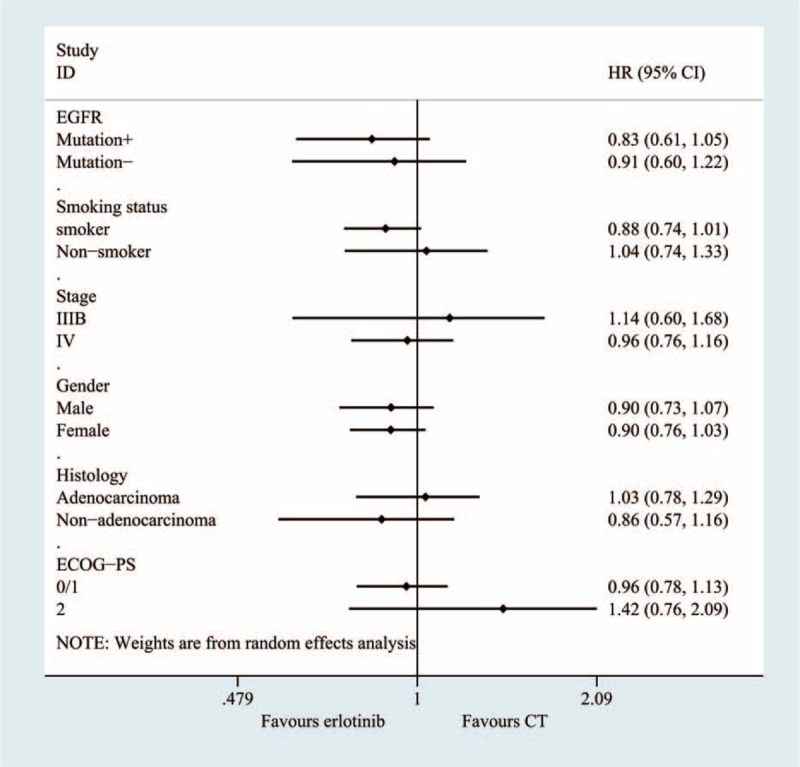
Subgroup and meta-regression analyses of the overall survival.

A subgroup analysis was adopted to determine the heterogeneity causes for the PFS (Figure 7) and OS (Figure 8) analyses. The effect sizes were similar between the subgroups, which were divided into 8 predefined subgroups according to gender, smoking status, histology and patient year, ECOG-PS, anatomic stage, and treatment status. No statistical significance was identified regarding treatment effect differences in the various subgroups, and the *P* values for gender, smoking status, histology and patient year, ECOG-PS, anatomic stage were 0.618, 0.443, 0.626, 0.395, 0.582, and 0.555 in PFS, respectively. The subgroup analysis based on EGFR mutation status appeared to be discordant, as the patients without EGFR mutations showed significantly prolonged PFS with chemotherapy (HR, 0.22; 95%CI = 0.15–0.30, *P* < 0.001). However, among the patients without EGFR mutations, conventional chemotherapy demonstrated decreased PFS (HR = 1.27; 95% CI = 1.04, 1.45) compared with erlotinib.

### Meta-Regression and Sensitivity Analysis

To investigate the effects of various study characteristics on HR estimates, a meta-regression analysis was conducted (only for the PFS results) by grouping the studies according to specific characteristics, such as pretrial EGFR test, sample size, trial phase, treatment status, and publication year. However, the univariate and multivariate meta-regression analyses did not detect a borderline significant association between PFS and pretrial EGFR test, or other characteristics^33,34^ (Table 3).

**TABLE 3:**
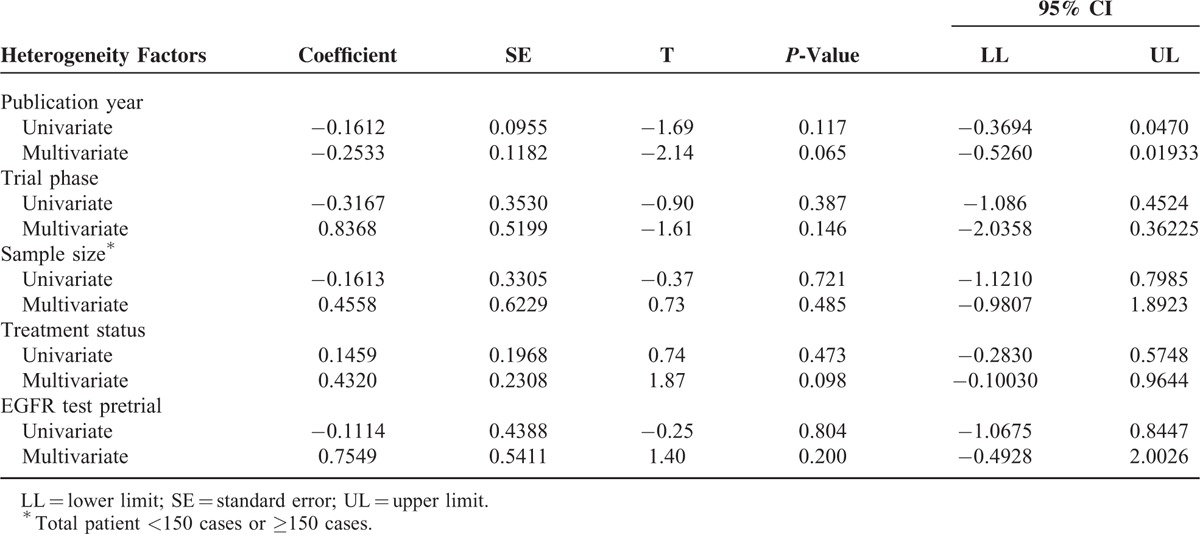
Univariate and Multivariate Meta-Regression Analyses of Potential Sources of Heterogeneity in PFS

**FIGURE 9:**
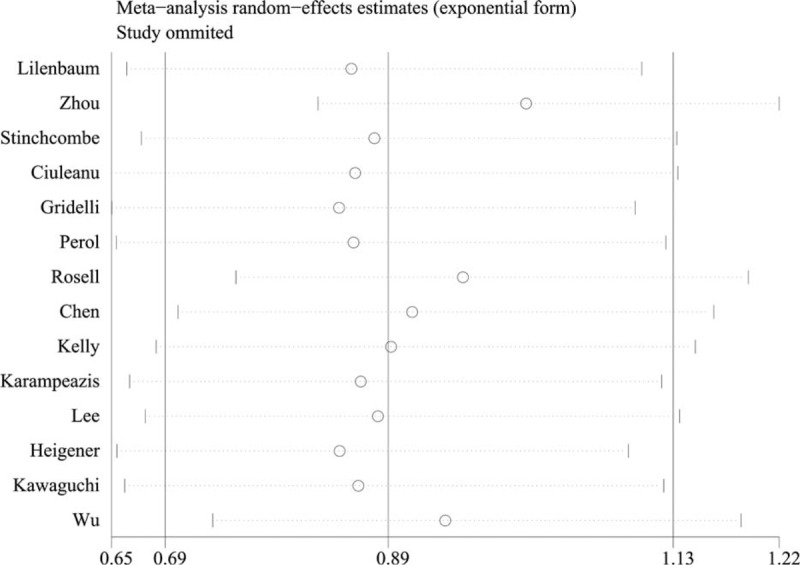
Sensitivity analysis result of progression-free survival.

The sensitivity analysis indicated that the pooled PFS results were affected by the exclusion of certain individual trials, specifically the trials of Zhou et al,^34,37^ Chen et al,^38^ and Rosell et al^33^ (Figure 9). However, the pooled OS results were not affected by the exclusion of individual trials.

### Publication Bias

A funnel plot was performed on all the included studies that investigated the OS and PFS efficacies to determine the publication bias from the literature. The analysis outcome showed asymmetry, which suggests that a publication bias possibly existed in the included trials. Begg and Egger tests were performed to quantitatively test the asymmetry of the funnel plot, and no bias was determined for the OS (*P*_Egger_ = 0.194, *P*_Begg_ = 0.194) and PFS rates (*P*_Egger_ = 0.066, *P*_Begg_ = 0.066).

### Level of Evidence

There were 4 efficacy outcomes in this meta-analysis. The OS and PFS rates were critical results, and the 1-year survival and ORR were important results. The quality of the evidence of each result is reported in Table 4.

**TABLE 4:**
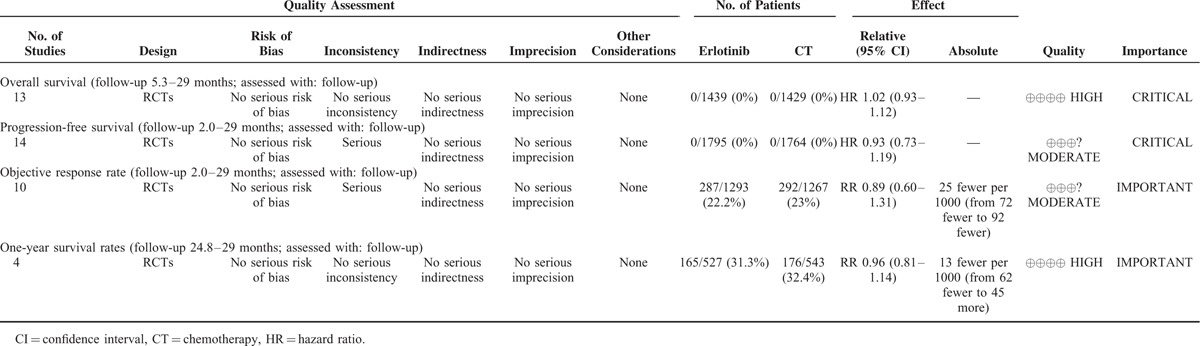
GRADE Profile Evidence of the Included Studies

## DISCUSSION

The pooled results of the meta-analysis that utilized 14 RCTs to compare erlotinib and chemotherapy treatment groups demonstrated that no significant difference existed regarding most outcomes, including the OS, PFS, 1-year survival and objective response rates. In this review, subgroup analyses were conducted according to EGFR status, drug condition, histology condition, length of illness and other factors. The subgroup analysis, which was based on EGFR mutations, suggested that erlotinib could greatly increase the PFS rate in patients with an EGFR mutation (HR, 0.22; 95% CI = 0.15–0.30, *P* < 0.0001). For patients without an EGFR mutation, the usage of erlotinib inversely varied with the PFS rate. In other words, the PFS time could increase with erlotinib use. The pooled results showed that erlotinib use was unrelated to OS among patients with and without an EGFR mutation. The sample size and mutation condition tests can explain most of the heterogeneity observed, according to the results of the sensitivity analysis and meta-regression. The sensitivity analysis using the leave-one-out method revealed that the heterogeneity was decreased to 67% when the 2 trials that evaluated mutation conditions were eliminated.

Several meta-analyses on EGFR-TKIs have been published in recent years, most of which employed trials with varying drug priorities. A majority of the published studies focused on efficacy, while the correlation between EGFR mutation and efficacy was reported in 4 meta-analyses.^11–14^ Additionally, of these meta-analyses, 3 focused on the relationship between the mutation type and efficacy; however, 1 study, which was published in JAMA,^13^ did not assess this correlation. Another major point that was not presented in all 4 of these meta-analyses is that the efficacy may be affected by sample size, the eligible patient age, treatment duration and other factors. Deficiencies in some published studies were managed by planning in the meta-analysis, in which some stage 2 clinical trials were included. The meta-analysis showed that no difference existed between stage 2 and 3 of the clinical drug groups. Importantly, the GRADE system was performed to assess the level of evidence summarized in the meta-analysis.

There are a number of limitations to this meta-analysis that need to be acknowledged. First, only English and Chinese language literature articles were considered in the analysis. If the search had been extended to include literature published in other languages, it is possible that additional relevant trials may have been identified. Second, on-going studies were ineligible for inclusion, although this meta-analysis included in 14 studies, but the sample is not very enough, some studies were small samples. Limitations in quality, even though most of the studies were of high quality, cannot be ignored, and the pooled results of this meta-analysis may have been slightly affected. Moreover, only a small number of trials met the subgroup analysis criteria, thus reducing the power of the analysis. Additionally, some parameters are not coming from the real data, we used the Tierney et al's^17^ formula to calculate the missing hazard rate, although our previous research used this method,^9^ but this formula might reduce the credibility of the analysis results. Furthermore, as the studies included in the meta-analysis were carried out in various countries, oncologists should carefully and judiciously assess the feasibility of applying the results to the clinical setting in China.

In conclusion, the present systematic review and meta-analysis suggested that erlotinib did not improve the ORR, PFS, OS, or the 1-year survival rate for whole patients with or without EGFR mutation test. Nevertheless, the subgroup analysis revealed that erlotinib did not affect the OS regardless of EGFR mutation status, however, the agent prolonged PFS in subjects with EGFR mutation, but not in those without EGFR mutation. The GRADE system suggested our evidences are of good quality, however, our finding partly relies on data from Kaplan–Meier curves by Engauge Digitizer (version 4.1), potentially subject to other bias, this conclusion should be interpreted cautiously, and thus this conclusion should be interpreted cautiously, and the meta-regression did not find significant association between PFS and characteristics. Therefore, high-quality and adequately powered RCTs for this subgroup patients are warranted.
